# Influence of spacer and milling strategy on CAD/CAM ceramic crown fit and load-bearing capacity

**DOI:** 10.4317/jced.63812

**Published:** 2026-04-25

**Authors:** Ricardo-Susin Schelbauer, Ana-Laura Rosa, Gabriela-de-Souza Balbinot, Fabricio-Mezzomo Collares, Marina-da-Rosa Kaizer, Carla-Castiglia Gonzaga

**Affiliations:** 1 School of Health Sciences, Graduate Program in Dentistry, Universidade Positivo (UP), Curitiba, PR, Brazil; 2 Dental Materials Laboratory, School of Dentistry, Federal University of Rio Grande do Sul, Porto Alegre, RS, Brazil; 3 Department of Restorative Sciences, School of Dentistry - University of Alabama at Birmingham (UAB), Birmingham, AL, USA

## Abstract

**Background:**

This study assessed the influence of virtual spacer (70, 110, 150 m) and milling strategy (Standard vs. Veneer) on marginal and internal fit, and load-bearing capacity of CAD/CAM ceramic crowns.

**Material and Methods:**

Sixty composite resin abutments were scanned, and feldspathic ceramic crowns (CEREC Blocs) were fabricated. Micro-computed tomography evaluated marginal and internal fit on sagittal and axial planes. Crowns were bonded before monotonic load-bearing capacity testing. Data were analyzed using two-way ANOVA and Tukey's test (=5%).

**Results:**

Marginal fit showed no significant differences among milling strategies or spacer settings, and values for all groups were within the clinically accepted range (~100 to 150 m). The virtual spacer, but not the milling strategy, significantly influenced the internal fit at specific sites on both sagittal and axial planes. The 150 m spacer produced larger internal gaps compared to the 70 m setting at the affected sites. Load-bearing capacity (ranging from 924 N to 1297 N) showed no significant differences among any conditions tested.

**Conclusions:**

The virtual spacer thickness influenced the internal fit of ceramic crowns at specific measurement sites, while the milling strategy did not. Neither factor significantly affected the immediate load-bearing capacity under monotonic loading. Marginal fit remained clinically acceptable across all conditions.

## Introduction

The long-term success of indirect esthetic restorations is critically dependent on factors such as esthetics, fracture resistance, and fit (1). Poor adaptation can lead to detrimental outcomes, including hypersensitivity, recurrent caries, and periodontal problems (2). While a cement gap of 100 to 120 m is widely considered clinically acceptable (3 , 4), an excessive internal misfit reduces fracture resistance. This occurs because the lower elastic modulus of the luting cement, compared to the ceramic, concentrates stress at the ceramic-cement interface, potentially leading to failure (5 - 7). The advent of CAD/CAM technology fundamentally digitized the management of cement space for indirect restorations. However, despite its critical role, no clear consensus exists regarding the optimal virtual spacer thickness to achieve an ideal fit in ceramic crowns (8). Research has extensively investigated the influence of virtual spacer settings on CAD/CAM ceramic crown fit across various indirect restorative materials. Studies evaluating restorations have suggested that lower settings (e.g., 30 m) may provide optimal fit, though acceptable ranges up to 80 m have been reported for CEREC and E4D CAD/CAM systems (5 , 8 , 9). Furthermore, some studies reported that varying spacer thicknesses between 50 m and 80 m had no significant effect on the fit of polymer-infiltrated ceramic crowns (10). This discrepancy emphasizes the need for further research to elucidate the clinical outcomes of different spacer thicknesses for CAD/CAM restorations. In addition to fit, the subsequent effect on mechanical performance has also been examined. Previous investigations using feldspathic ceramic crowns concluded that varying the spacer thickness (across ranges such as 30 to 150 m) did not significantly affect the load-bearing capacity (11 , 12). A significant limitation of CAD/CAM systems is their compromised ability to accurately reproduce poorly designed or irregular preparation features, primarily due to the oversized milling burs. Typical diamond burs used in common CAD/CAM systems have large diameters (1.3 to 1.5 mm) and cannot precisely mill defects smaller than the bur diameter (13). These inaccuracies can result in overmilling (creating a marginal gap) or undermilling (hindering complete seating) (13). To mitigate these limitations, the CEREC system introduced two distinct milling strategies: Standard and Veneer (14). The Standard milling strategy intentionally uses overmilling, where the software removes extra ceramic material in detected interference areas to ensure passive seating. This process typically utilizes a flat-topped step bur and aims for a guaranteed fit (14). Conversely, the Veneer milling strategy forgoes overmilling, meaning potential internal interferences are not eliminated. This approach, using a cylinder-pointed bur with a smaller tip thickness, requires more precise preparations but is proposed to optimize surface finish and adaptation, particularly for thin restorations like veneers (13 , 14). Although the influence of spacer settings on marginal fit is well established, there is limited information regarding how different milling strategies affect the mechanical performance of the crowns. Furthermore, it is unclear how the interaction between these milling parameters and cement space settings influences the final load-bearing capacity of the restorations. Therefore, the present study aimed to assess the influence of virtual spacer settings (70 m, 110 m, and 150 m) and milling strategies (Standard and Veneer) on the marginal and internal adaptation and load-bearing capacity of CAD/CAM feldspathic single crowns. The following null hypotheses were tested: i) spacer thickness, regardless of the milling strategy, would not affect the marginal and internal fit or the load-bearing capacity of ceramic crowns; and ii) the milling strategy, irrespective of spacer thickness, would not interfere with the marginal and internal fit or the load-bearing capacity of ceramic crowns.

## Material and Methods

**Figure 1:**
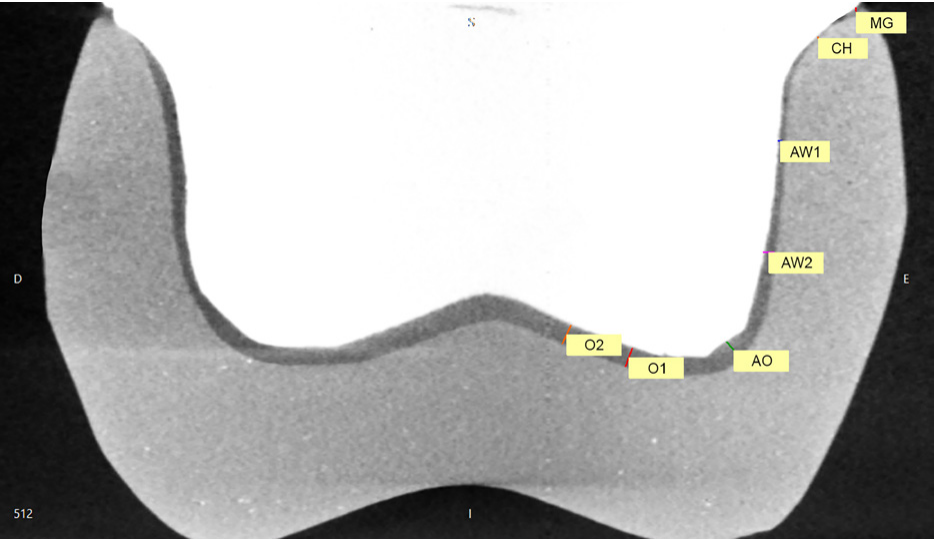
Marginal and internal fit assessment points on sagittal plane. Marginal gap (MG); misfit in chamfer area (CH); misfit at cervical third of axial wall (AW1); misfit at middle third of axial wall (AW2); misfit at axial-occlusal angle (AO); misfit in occlusal area (O1), at the end of the first third of the inner occlusal surface between the AO angle and the central groove; and misfit in the occlusal area (O2), determined at the end of the second third of the inner occlusal surface between the AO angle and the central groove.

To evaluate the influence of the variables mentioned above, this study followed a factorial experimental design (3 x 2), considering spacer thickness (70 m, 110 m, and 150 m) and milling strategy (Standard and Veneer) as the primary factors. The outcomes were defined as marginal/internal fit and load-bearing capacity. A standardized master die representing a 5 mm tall mandibular second premolar was prepared to receive an all-ceramic crown. The preparation featured an axial wall convergence of 8o to 12o, a uniform occlusal and axial reduction of 1 mm, and a chamfer finish line. Impressions were taken using a heavy-bodied condensation silicone (Zetalabor; Zhermack) to create 60 individual abutments. To simulate the natural tooth structure, these abutments were fabricated with composite resin (Filtek Z100; 3M) due to its elastic modulus (18 GPa) (15 , 16), which is similar to that of human dentin (16 to 18 GPa) (17). The elastic modulus of the supporting structure (abutment + cement) is known to significantly influence restoration fracture resistance (6 , 7 , 18). Abutments were scanned (Primescan; Dentsply Sirona), and full crowns were designed using the Biogeneric Individual design mode in the CEREC 5.02 software (Dentsply Sirona). The design stipulated a uniform crown thickness of 1 mm across all surfaces, including the center of the occlusal fossa. Sixty feldspathic ceramic crowns (CEREC Blocs; Dentsply Sirona) were milled (CEREC MCXL; Dentsply Sirona) and allocated to six experimental groups (n=10 per group) based on a combination of: 1) milling strategy (Standard or Veneer) and 2) virtual spacer thickness setting (70 m, 110 m, or 150 m). The specific milling tools were the step bur 12S for the Standard strategy and the cylinder-pointed bur 12S for the Veneer strategy. All virtual margins were set to 150 m in the software to define the cementation limit. All laboratory steps were performed by a single, experienced operator with expertise in prosthetic and digital dentistry to ensure standardization. For fit analysis, a subset of three abutment/crown sets per experimental group was randomly selected for micro-computed tomography (CT) scanning (MicroCT.SMX-90 CT; Shimadzu Corp., Kyoto, Japan). Crowns were passively seated onto the abutments using a light-body addition silicone (Futura AD; DFL, Rio de Janeiro, Brazil) to simulate the luting procedure without cementation pressure. Scans were acquired at 360 degrees, 90 kV, 100 mA, using a 1 mm aluminum filter. Images were reconstructed using the inspeXio SMX-90CT software (Shimadzu Corp.) with a voxel size of 12 m and a resolution of 1024 × 1024 pixels, resulting in 541 images per sample. The CT images were 3D-reconstructed and assessed using the InVesalius software (Centro de Tecnologia da Informação Renato Archer, Brazil, &lt;http://www.cti.gov.br/invesalius/&gt;). Marginal and internal fit were manually measured as the distance between the inner surface of the crown and the external surface of the abutment at fifteen distinct measurement sites. These sites were distributed as follows: Sagittal (longitudinal) plane (seven points) (Fig. 1): Measurements were taken at the center, mesial, and distal aspects of the crown.

1

**Figure 2:**
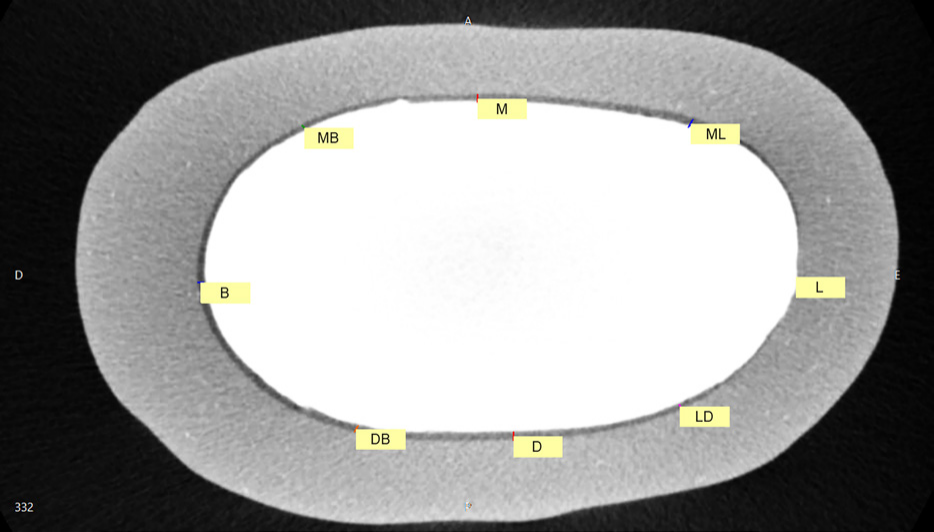
Internal fit assessment points on axial plane. Center of buccal (B), mesiobuccal (MB), mesial (M), mesiolingual (ML), lingual (L), distolingual (DL), distal (D), and distobuccal (DB) areas.

The parameters evaluated were: marginal gap (MG); misfit in the center of the chamfer area (CH); misfit at the cervical third of the axial wall (AW1); misfit at the middle third of the axial wall (AW2); misfit at the axial-occlusal angle (AO); and misfit in the occlusal area at the end of the first and second thirds of the inner surface (O1 and O2). Axial plane (eight points) (Fig. 2): Measurements were determined at the cervical, middle, and occlusal thirds, focusing on the center of the: buccal (B), mesiobuccal (MB), mesial (M), mesiolingual (ML), lingual (L), distolingual (DL), distal (D), and distobuccal (DB) surfaces.

2

To ensure the reliability of the fit assessment, all measurements were performed by a single, trained operator. The protocol was based on previously established methodologies for sagittal analysis (19 , 20), which were further expanded in this study to include an assessment of the axial plane. Pre-defined anatomical landmarks in both planes strictly guided point selection to ensure a comprehensive and reproducible evaluation while minimizing selection bias. Following image acquisition, the ceramic crowns were carefully detached from the abutments. The crowns were cleaned by immersion in 70% ethanol in an ultrasonic bath for 3 min. The intaglio surface of the crowns was then etched with 10% hydrofluoric acid (Condac Porcelana 10%; FGM, Joinville, Brazil) for 60 s, copiously rinsed, dried, and coated with a silane coupling agent (Prosil; FGM) for 60 s. The composite resin abutment surfaces were subsequently etched with 37% phosphoric acid (FGM) for 15 s, thoroughly rinsed, and dried. The crowns were luted to the abutments using a self-adhesive resin cement (Maxcem Elite; Kerr, Orange, CA, USA). To ensure a standardized cement film thickness across all specimens and minimize confounding variables, each specimen was subjected to a constant vertical seating load of 5 kg applied by a specific loading device. Excess cement was immediately removed, and the cement was light-cured according to the manufacturer's instructions. The specimens were then stored at 37oC and 100% relative humidity for 7 days to ensure complete post-cure polymerization and full hydration of the cement before mechanical testing. The specimens were subjected to a single static, monotonic load test, specifically a Hertzian-type indentation test, using a universal testing machine (DL 2000; EMIC, São José dos Pinhais, Brazil). A vertical occlusal load was applied along the specimen's long axis by a tungsten-carbide spherical indenter (3-mm radius), positioned centrally on the occlusal fossa of the crown. The test was carried out at a crosshead speed of 0.5 mm/min. The load-bearing capacity (N) was recorded as the highest load achieved before a load drop of at least 25%, indicating bulk failure. The cracked and/or fractured specimens and fragments were collected, and the failure mode was visually assessed and classified as follows: cracking or chipping, hoop fracture in the cervical region, splitting of the crown into two fragments through the central groove, and catastrophic fracture. The sample size for the mechanical test (n=10) and for the micro-CT analysis (n=3) was determined based on previous literature and the specific requirements of each methodology. For the micro-CT, the high number of measurement points per specimen (15 points) compensated for the smaller number of crowns, ensuring a representative mapping of the internal and marginal fit. For data analysis, the marginal and internal fit measurements were assessed separately for each of the 15 measurement sites. The normality and homoscedasticity of the data for each site were assessed using the Shapiro-Wilk and Levene's tests, respectively. To address the primary aim, the influence of the two independent factors (milling strategy and virtual spacer) on the outcomes (fit and load) was analyzed using two-way analysis of variance (ANOVA). Given that multiple comparisons were performed across numerous measurement sites, a strategy was implemented to control the familywise type I error rate. For comparisons between the spacer groups and the milling strategies, post-hoc analysis was performed using Tukey's HSD test (=5%). To evaluate the discrepancy between the virtual design and the physical outcome, the mean fit values from the axial wall (AW1 and AW2) and occlusal surface (O1 and O2) measurement sites were pooled by group and compared against the respective virtual spacer parameter values (70 m, 110 m, or 150 m) using Dunnett's test. The fracture load data were also analyzed statistically by two-way ANOVA. A significance level of =0.05, power &gt;80% was set for all analyses.

## Results

The findings for marginal and internal fit at the seven specific sites assessed on the sagittal plane are shown in Table 1.

Table 1

For all measurement sites and groups, the data met the assumptions of normality (p&gt;0.298) and homoscedasticity (p&gt;0.114). The two-way ANOVA revealed no statistically significant differences for the milling strategy or the interaction (milling strategy × spacer) for any of the evaluated sites. Significant differences attributable only to the virtual spacer setting were found at the cervical third of the axial wall (AW1, p=0.026), the middle third of the axial wall (AW2, p=0.021), and at the axial-occlusal angle (AO, p=0.045). At these specific sites, crowns fabricated with the 150 m virtual spacer setting exhibited a statistically larger misfit when compared with the 70 m setting. The marginal fit values (MG) for all groups remained statistically comparable and fell within the accepted clinical range (Table 1). The findings for internal fit at the eight sites assessed at the middle third on the axial plane are shown in Table 2.

Table 2

All data met the assumptions of normality (p&gt;0.287) and homoscedasticity (p&gt;0.208). The two-way ANOVA revealed no statistically significant differences for the milling strategy or the interaction (milling strategy × spacer) across any of the evaluated sites. Significant differences were identified, related solely to the virtual spacer setting, at the lingual (L, p&lt;0.001), distolingual (DL, p=0.005), buccal (B, p=0.027), and mesiobuccal (MB, p=0.024) sites. Post-hoc analysis confirmed that at these measurement sites, the 150 m virtual spacer setting resulted in a statistically larger internal misfit when compared with the 70 m setting. The comparison of the mean internal fit obtained experimentally with the virtual spacer settings predefined during the crown design was performed using Dunnett's test (Table 3).

Table 3

This analysis aimed to determine the discrepancy between the planned and the achieved luting space. Significant statistical differences between the measured mean internal fit and the pre-set virtual value were observed exclusively for the 70 m spacer setting on the inner occlusal surface, regardless of the milling strategy used. This suggests an inaccuracy in the reproduction of the smaller, 70 m virtual space in the occlusal region. In contrast, no significant differences were found for the 110 m and 150 m spacers on the inner occlusal surface, indicating a greater fidelity to the virtual design at these larger settings. Furthermore, the mean internal fit measured on the axial wall showed no statistically significant differences from the respective virtual spacer settings, irrespective of the milling strategy or spacer value used. The load-bearing capacity data, detailed in Table 4, met the assumptions of normality (p=0.289) and homoscedasticity (p=0.982).

Table 4

**Figure 3:**
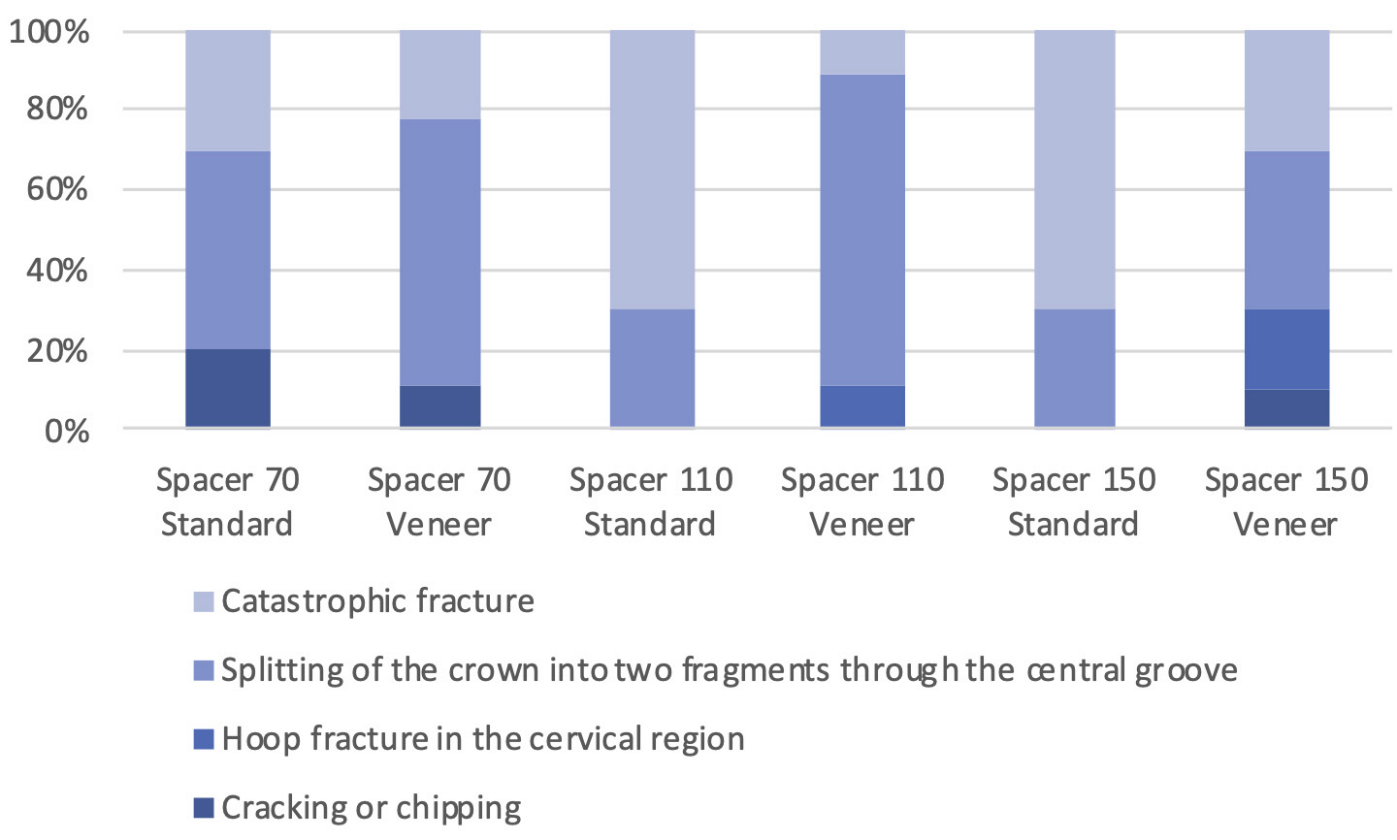
Cumulative frequencies of failure modes according to milling strategy and spacer.

The two-way ANOVA revealed no statistically significant differences in load-bearing capacity for the milling strategy (p=0.291), the virtual spacer setting (p=0.074), or their interaction (p=0.247). The load-bearing capacity for all groups was high, ranging from 924 N to 1297 N, demonstrating that neither the milling approach nor the virtual spacer setting significantly influenced the resistance to single static failure. Following the Hertzian Indentation test, specimens were classified according to failure mode (Fig. 3).

3

Most failures (70% or higher) were catastrophic, occurring predominantly as crown splitting into two fragments through the central groove or as complete catastrophic fracture. A smaller percentage (up to 30%) of failures were classified as less severe, including cracking or chipping and hoop fracture in the cervical region.

## Discussion

The first null hypothesis was rejected for internal fit but accepted for marginal fit and load-bearing capacity. The 150 m virtual spacer setting resulted in a statistically larger internal misfit when compared to the 70 m setting. This influence was site-specific and did not compromise the overall marginal fit or the mechanical resistance of the restorations. The second null hypothesis was accepted. The results showed no statistically significant differences between the Veneer and Standard milling strategies for any measured outcome. This may be attributed to two primary mechanisms: either the subtle differences in bur geometry and milling parameters were insufficient to create a measurable change in fit within the CT resolution, or the inherent variability of the overall CAD/CAM fabrication process masked any minor influence of the milling strategy. The CEREC MCXL milling machine operates with a 3+1 axis system, utilizing three simultaneous rotary motors and one linear motor. Despite the technical differences in bur sequencing, where the Standard strategy uses a diamond step bur for the inner surface and the Veneer strategy incorporates a linear axis rotation, no significant effect was observed on marginal and internal fit, or load-bearing capacity. This lack of significant difference in fit is consistent with other studies that compared different milling protocols, finding minimal impact on the internal adaptation of ceramic crowns (21). The limited research available on the effect of milling strategies makes assessing their influence on load-bearing capacity challenging. To maximize data reliability, several steps were taken: A standardized protocol was used for all mechanical testing, ensuring consistent loading direction and crosshead speed. Furthermore, consistent cementation and careful inspection of abutment uniformity minimized variability. However, inherent variation is expected in load-bearing tests involving brittle ceramic materials (22 , 23). The observed range in load-bearing capacity may arise from minor variations in crown thickness, subtle differences in the bonding interface, or inherent material variability. While micro-CT analysis provided detailed internal fit data, it remains plausible that variations below the 12 m voxel resolution of the CT, or in areas not captured by the specific measurement sites, could have contributed to the observed range in load-bearing capacity. It is important to acknowledge the limitations inherent in the mechanical testing protocol. The use of a single static failure mode does not incorporate the effects of thermocycling or mechanical fatigue simulation. Consequently, while our results confirm that neither the milling strategy nor the virtual spacer setting influences the immediate resistance to fracture, these findings cannot be extrapolated to predict long-term clinical performance or degradation under simulated oral aging conditions. Future studies incorporating cyclic loading are necessary to assess the clinical relevance of these fabrication parameters on the longevity of the crowns. The selection of measurement sites for marginal and internal fit was strategically based on the distinct action of the milling burs on the inner surface of the restorations, aiming to provide a detailed assessment of the fit discrepancy. Although the number of measurement points in the literature varies widely (ranging from 4 to over 3,500 in systematic reviews) (24), relying on only a few points offers limited insight. Conversely, an excessive number of points can complicate the analysis. A more reliable assessment was ensured by selecting 15 assessment points, thereby balancing the need for comprehensive data with analytical feasibility. The sagittal plane is standard in fit analysis, providing a comprehensive observation from the margin to the occlusal surface. The points chosen on the axial wall and axio-occlusal angle are particularly relevant due to their role in crown retention and their susceptibility to fabrication discrepancies. The inclusion of the axial plane, however, provided an advantageous perspective by evaluating the circumferential fit and the overall internal adaptation along the entire perimeter of the cement line. Regarding clinical relevance, the clinically acceptable marginal fit for indirect restorations shows considerable variation in the literature, generally ranging from 20 m to 200 m (3 , 24 - 27). Similarly, reported internal fit values range broadly, from approximately 23 m to 230 m in systematic reviews (24 , 28). All marginal and internal fit values obtained in the present study are consistently in line with these reported ranges and are considered clinically acceptable. The wide variability in milling parameters across CAD/CAM systems, particularly the space reserved for cement, emphasizes the lack of consensus in the field. These settings are frequently established empirically, relying on user experience and clinical observation. It is well-documented that the virtual spacer significantly influences fit (5 , 29 , 30). Specifically, smaller spacer settings can lead to premature crown contact with the preparation surface, hindering the extrusion of excess luting cement and consequently increasing marginal and internal discrepancies (5 , 29 , 30). Furthermore, while small spacers (e.g., 30 to 50 m) may be viable in ideal laboratory conditions, their clinical management poses significant challenges for achieving full crown seating (30). Although a marginal misfit up to 120 m is generally accepted (3 , 4), the present study explored a wider spectrum of virtual spacer settings (70 m, 110 m, and 150 m) for comprehensive analysis. The primary reason for including the largest value (150 m), despite its acknowledged controversial clinical applicability, was to assess the sensitivity of the internal fit to substantial deviations from the ideal. This value, while unlikely to be intentionally selected by clinicians, could occur because of fabrication errors or procedural variations, thus offering valuable information regarding the potential consequences of such inaccuracies. The 70 m setting served as a conservative baseline for comparison. Furthermore, the chosen progressive increments facilitated a clearer analysis of the relationship between spacer thickness and the resultant fit. Therefore, the inclusion of the 150 m spacer was a methodological necessity to explore the full range of potential discrepancies in the CAD/CAM workflow, rather than an endorsement for its routine clinical use. In the present study, the larger virtual spacer setting (150 m) consistently resulted in larger internal discrepancies compared to the 70 m setting. This increased misfit primarily occurred in the occlusal region, specifically at the axio-occlusal angle and the inner occlusal surface. This finding highlights one of the most critical areas for potential seating inaccuracy. The large internal discrepancies observed in these occlusal areas are likely exacerbated by the "plunger effect" during cementation (31). This effect, caused by the hydraulic pressure of the cement confined between the restoration and the preparation, can prevent complete seating and consequently increase the internal misfit in the load-bearing and retentive zones. The CT findings on the internal fit of axial and occlusal walls showed that the measured luting space frequently exceeded the virtual space predefined in the software. This observation is consistent with the literature, where measured axial and occlusal misfits were found to be substantially greater than the 60-m and 100-m virtual spacers tested in comparable research (8). The fact that the milling strategy did not significantly influence the marginal or internal fit in most measurement sites is supported by findings from studies comparing different protocols (21). However, other research indicates that the use of finer milling strategies consistently improves marginal fit (32). This contrast suggests that the influence of the milling strategy is highly dependent on the specific CAD/CAM system and material used, a finding that emphasizes the importance of the present study in defining the specific parameters for feldspathic ceramic crowns (32). To formally assess the discrepancy, Dunnett's test was utilized to compare the mean measured fit with the respective virtual spacer settings (70 m, 110 m, and 150 m). The results demonstrated a statistically significant discrepancy only for the 70-m spacer on the inner occlusal surface, regardless of the milling strategy. In this critical area, the measured misfit was 128% (Standard) to 139% (Veneer) higher than the planned value. For the larger spacers (110 m and 150 m) on the occlusal surface, while not statistically significant, the measured values were still consistently 40% to 86% higher than predefined. In contrast, the mean fit on the axial wall showed no statistically significant difference from the virtual settings for any group, and measured values were 10% to 51% lower than the predefined space. While the present study demonstrates that the virtual spacer thickness significantly affects internal fit, with the 150 m setting consistently exhibiting greater misfit, the statistical differences observed may not necessarily translate into a clinically significant increase in the risk of microleakage or cement washout. Therefore, further research, particularly long-term clinical studies, is essential to definitively assess the clinical influence of these measured differences in internal fit. It is worth noting that a standardized full crown preparation was selected for this study to ensure maximum standardization and comparability with established CAD/CAM literature. While more complex geometries, such as onlays or veneers, are clinically relevant, they introduce additional geometric variables that could mask the specific effects of spacer settings and milling strategies. Nevertheless, this study has limitations typical of in vitro designs and the results should be interpreted considering that: i) although composite resin dies allowed for the standardization of the substrate's elastic modulus, they do not account for the natural variability of teeth or the stress distribution provided by the periodontal ligament; ii) the absence of thermocycling and cyclic fatigue means these immediate results do not reflect the long-term degradation of the ceramic or the adhesive interface; iii) the use of static compressive loading, while useful for initial material screening and comparing fabrication parameters, does not replicate the complex dynamic forces and cyclic fatigue that characterize long-term clinical fracture behavior; and iv) the focus on specific spacer values, milling strategies, and a single ceramic system may limit the transferability of the results to other materials or different CAD/CAM ecosystems and software defaults. Therefore, while these findings provide important data on the mechanical behavior and fit of the tested restorations, they should be interpreted with caution when translated to different clinical workflows and material combinations. CAD/CAM feldspathic ceramic crowns are frequently used for esthetic and time-efficient single-visit restorations in contemporary clinical practice, which makes the selection of milling parameters a critical clinical decision. The findings of the present study simplify this decision by demonstrating that neither the milling strategy nor the spacer setting affected the load-bearing capacity of the crowns, and the milling strategy had no significant influence on fit. The overall marginal fit for all groups remained within clinical acceptance limits, suggesting both milling strategies are viable, though the selection of a smaller spacer is recommended for optimizing internal adaptation.

## Conclusions

The load-bearing capacity of the CAD/CAM feldspathic ceramic crowns was not influenced by either the virtual spacer setting or the milling strategy (Standard vs. Veneer). Furthermore, the milling strategy showed no influence on the marginal or internal fit of the restorations across any spacer group. However, the spacer thickness was a determining factor for internal adaptation: the 150-m virtual spacer setting consistently resulted in larger internal discrepancies when compared to the 70-m setting. Despite these measurable differences, the overall marginal fit of all crown groups remained within the established range of clinical acceptability.

## Figures and Tables

**Table 1:** Table Means and standard deviations for marginal and internal fit in the central groove on the sagittal plane.

Assessment point	Milling strategy	70 Î¼m	110 Î¼m	150 Î¼m	Total
Marginal gap (MG)	Standard	129.1 ± 60.9	114.3 ± 55.8	82.3 ± 20.0	108.6 ± 47.3A
	Veneer	89.7 ± 30.9	156.6 ± 72.6	56.0 ± 31.7	100.8 ± 61.5A
	Total	109.4 ± 48.3a	135.5 ± 62.4a	69.2 ± 27.7a	
Chamfer (CH)	Standard	61.1 ± 21.5	23.8 ± 18.8	66.5 ± 39.5	50.5 ± 31.6A
	Veneer	39.9 ± 36.2	60.4 ± 37.1	32.1 ± 11.3	44.1 ± 29.4A
	Total	50.5 ± 29.1a	42.1 ± 33.1a	49.3 ± 32.1a	
Axial wall 1 (AW1)	Standard	54.4 ± 7.1	85.3 ± 53.2	110.5 ± 25.0	83.4 ± 38.3A
	Veneer	50.1 ± 38.2	46.3 ± 25.7	109.8 ± 31.4	68.7 ± 41.6A
	Total	52.3 ± 24.7a	65.8 ± 43.0ab	110.2 ± 25.4b	
Axial wall 2 (AW2)	Standard	39.4 ± 16.7	113.5 ± 77.2	108.4 ± 13.3	87.1 ± 53.8A
	Veneer	42.8 ± 28.1	62.4 ± 40.7	126.5 ± 31.2	77.3 ± 47.9A
	Total	41.1 ± 20.7a	88.0 ± 61.9ab	117.5 ± 23.6b	
Axio-oclusal angle (AO)	Standard	87.9 ± 12.7	145.9 ± 101.8	155.1 ± 41.8	129.7 ± 63.7A
	Veneer	84.1 ± 31.9	178.6 ± 23.4	146.2 ± 33.7	136.3 ± 49.0A
	Total	86.0 ± 21.8a	162.2 ± 68.4b	150.7 ± 34.3b	
Occlusal area 1 (O1)	Standard	171.3 ± 21.1	187.6 ± 31.1	254.3 ± 81.4	204.4 ± 58.8A
	Veneer	167.0 ± 37.4	204.7 ± 26.0	203.7 ± 61.5	191.8 ± 42.5A
	Total	169.2 ± 27.3a	196.1 ± 27.3a	229.0 ± 70.2a	
Occlusal area 2 (O2)	Standard	147.4 ± 25.0	189.1 ± 60.4	225.5 ± 69.1	187.4 ± 58.4A
	Veneer	167.2 ± 16.5	205.5 ± 23.8	215.7 ± 53.2	196.1 ± 37.5A
	Total	157.3 ± 21.8a	197.3 ± 42.0a	220.6 ± 55.4a	

*Values followed by the same uppercase letters in the columns are statistically similar (p > 0.05). Values followed by the same lowercase letters in the rows are statistically similar (p > 0.05).

**Table 2:** Table Means and standard deviations of internal fit for the points assessed at the middle third on the axial plane.

Assessment point	Milling strategy	70 Î¼m	110 Î¼m	150 Î¼m	Total
Mesial (M)	Standard	68.2 ± 14.0	75.6 ± 17.5	93.8 ± 51.0	79.2 ± 30.1A
	Veneer	80.6 ± 68.4	104 ± 40.0	129.7 ± 36.0	104.8 ± 48.4A
	Total	74.4± 44.7a	89.8 ± 31.7a	111.7 ± 44.1a	
Mesiolingual (ML)	Standard	71.1 ± 23.5	71.5 ± 14.5	55.6 ± 30.7	66.0 ± 22.1A
	Veneer	82.8 ± 56.3	77.6 ± 50.0	109.6 ± 60.0	90.0 ± 50.4A
	Total	76.9 ± 39.1a	74.6 ± 33.1a	82.6 ± 51.9a	
Lingual (L)	Standard	30.8 ± 21.5	96.7 ± 11.7	84.7 ± 30.8	70.8 ± 36.2A
	Veneer	34.4 ± 14.8	56.8 ± 15.2	133.1 ± 23.9	74.7 ± 47.6A
	Total	32.6 ± 16.6a	76.7 ± 25.0b	108.9 ± 36.2b	
Distolingual (DL)	Standard	35.3 ± 17.2	55.5 ± 34.8	100.6 ± 23.9	63.8 ± 36.6A
	Veneer	31.5 ± 11.6	29.4 ± 15.9	96.9 ± 53.2	52.6 ± 43.7A
	Total	33.4 ± 13.2a	42.5 ± 28.1a	98.7 ± 36.3b	
Distal (D)	Standard	38.2 ± 46.7	62.5 ± 21.0	100.8 ± 54.1	67.1 ± 46.2A
	Veneer	46.0 ± 11.1	72.5 ± 15.8	63.1 ± 29.3	60.6 ± 21.0A
	Total	42.1 ± 30.6a	67.5 ± 17.5a	81.9 ± 44.0a	
Distobuccal (DB)	Standard	42.2 ± 31.8	44.8 ± 13.6	115.3 ± 64.6	67.4 ± 51.3A
	Veneer	56.1 ± 19.5	57.6 ± 26.3	62.8 ± 23.2	58.8 ± 20.3A
	Total	49.1 ± 24.8a	51.2 ± 20.0a	89.0 ± 52.1a	
Buccal (B)	Standard	43.2 ± 22.2	47.8 ± 32.0	123.6 ± 37.0	71.6 ± 47.4A
	Veneer	42.9 ± 13.2	58.1 ± 44.9	69.7 ± 28.3	56.9 ± 29.7A
	Total	43.1 ± 16.4a	53.0 ± 35.3ab	96.7 ± 41.7b	
Mesiobuccal (MB)	Standard	62.6 ± 5.8	75.9 ± 14.3	81.6 ± 52.9	73.3 ± 28.8A
	Veneer	28.7 ± 24.4	100.1 ± 47.4	125.5 ± 18.6	84.7 ± 51.8A
	Total	45.6 ± 24.4a	88.0 ± 34.0ab	103.5 ± 42.8b	

*Values followed by the same uppercase letters in the columns are statistically similar (p > 0.05). Values followed by the same lowercase letters in the rows are statistically similar (p > 0.05).

**Table 3:** Table Means and standard deviations of misfit (in μm) on axial walls (AW1 and AW2) and occlusal inner surface (O1 and O2). P-value corresponds to the mean values compared with those used as reference (predefined value) in the software for crown design, according to Dunnett’s test.

Milling strategy	Spacer (Î¼m)	Mean axial wall (Î¼m)	p-value	Mean occlusal inner surface (Î¼m)	p-value
Standard	70	46.9 ± 14.1	0.605	159.4 ± 24.5	0.016
	110	99.4 ± 61.3	0.974	188.3 ± 43.0	0.111
	150	109.5 ± 17.9	0.272	239.9 ± 69.4	0.349
Veneer	70	46.5 ± 30.3	0.595	167.1 ± 25.9	0.010
	110	54.4 ± 31.7	0.512	205.1 ± 22.3	0.052
	150	118.1 ± 29.3	0.425	209.7 ± 51.8	0.606

3

**Table 4:** Table Means and standard deviations of load-bearing capacity according to the milling strategy and spacer.

Spacer (Î¼m)	Load-bearing capacity (N)	
	Standard	Veneer
70	924 ± 320	1142 ± 227
110	1088 ± 309	1204 ± 300
150	1297 ± 300	1206 ± 276

4

## Data Availability

The datasets used and/or analyzed during the current study are available from the corresponding author.

## References

[1] Contrepois M, Soenen A, Bartala M, Laviole O (2013). Marginal adaptation of ceramic crowns: a systematic review. J Prosthet Dent.

[2] Srimaneepong V, Heboyan A, Zafar MS, Khurshid Z, Marya A, Fernandes GVO, Rokaya D (2022). Fixed prosthetic restorations and periodontal health: a narrative review. J Funct Biomater.

[3] McLean JW, von Fraunhofer JA (1971). The estimation of cement film thickness by an in vivo technique. Br Dent J.

[4] Sorensen JA (1990). A standardized method for determination of crown margin fidelity. J Prosthet Dent.

[5] Nakamura T, Dei N, Kojima T, Wakabayashi K (2003). Marginal and internal fit of CEREC 3 CAD/CAM all-ceramic crowns. Int J Prosthodont.

[6] El Zhawi H, Kaizer MR, Chughtai A, Moraes RR, Zhang Y (2016). Polymer infiltrated ceramic network structures for resistance to fatigue fracture and wear. Dent Mater.

[7] Yan J, Kaizer MR, Zhang Y (2018). Load-bearing capacity of lithium disilicate and ultra-translucent zirconias. J Mech Behav Biomed Mater.

[8] Mously HA, Finkelman M, Zandparsa R, Hirayama H (2014). Marginal and internal adaptation of ceramic crown restorations fabricated with CAD/CAM technology and the heat-press technique. J Prosthet Dent.

[9] Shim JS, Lee JS, Lee JY, Choi YJ, Shin SW, Ryu JJ (2015). Effect of software version and parameter settings on the marginal and internal adaptation of crowns fabricated with the CAD/CAM system. J Appl Oral Sci.

[10] Dauti R, Lilaj B, Heimel P, Moritz A, Schedle A, Cvikl B (2020). Influence of two different cement space settings and three different cement types on the fit of polymer-infiltrated ceramic network material crowns manufactured using a complete digital workflow. Clin Oral Investig.

[11] Sagsoz NP, Yanikoglu N (2018). Evaluation of the fracture resistance of computer-aided design/computer-aided manufacturing monolithic crowns prepared in different cement thicknesses. Niger J Clin Pract.

[12] Zahran M (2020). Effect of adhesive gap setting on fracture resistance of all-ceramic crowns. Open Dent J.

[13] Renne W, Wolf B, Kessler R, McPherson K, Mennito AS (2015). Evaluation of the marginal fit of CAD/CAM crowns fabricated using two different chairside CAD/CAM systems on preparations of varying quality. J Esthet Restor Dent.

[14] Zimmermann M, Valcanaia A, Neiva G, Mehl A, Fasbinder D (2018). Influence of different CAM strategies on the fit of partial crown restorations: a digital three-dimensional evaluation. Oper Dent.

[15] Kim JW, Kim JH, Thompson VP, Zhang Y (2007). Sliding contact fatigue damage in layered ceramic structures. J Dent Res.

[16] Zhang Y, Kim JW, Bhowmick S, Thompson VP, Rekow ED (2009). Competition of fracture mechanisms in monolithic dental ceramics: flat model systems. J Biomed Mater Res B Appl Biomater.

[17] Kinney JH, Marshall SJ, Marshall GW (2003). The mechanical properties of human dentin: a critical review and re-evaluation of the dental literature. Crit Rev Oral Biol Med.

[18] Shembish FA, Tong H, Kaizer M, Janal MN, Thompson VP, Opdam NJ, Zhang Y (2016). Fatigue resistance of CAD/CAM resin composite molar crowns. Dent Mater.

[19] Cunali RS, Saab RC, Correr GM, Cunha LFD, Ornaghi BP, Ritter AV, Gonzaga CC (2017). Marginal and internal adaptation of zirconia crowns: a comparative study of assessment methods. Braz Dent J.

[20] Saab RC, da Cunha LF, Gonzaga CC, Mushashe AM, Correr GM (2018). Micro-CT analysis of Y-TZP copings made by different CAD/CAM systems: marginal and internal fit. Int J Dent.

[21] Tribst JPM, Hosseini F, Pilecco RO, Serrano CM, Kleverlaan CJ, Dal Piva AMO (2024). The influence of extra-fine milling protocol on the internal fit of CAD/CAM composite and ceramic crowns. Materials (Basel).

[22] Paque PN, Gantner C, Matzener KJ, Özcan M, Ioannidis A (2024). Load-bearing capacity, internal accuracy and time-efficiency of heat-pressed, milled and 3D-printed lithium disilicate ultra-thin occlusal veneers. Dent Mater.

[23] Mörikofer N, Benic GI, Park JM, Özcan M, Hüsler J, Ioannidis A (2021). Relationship between internal accuracy and load-bearing capacity of minimally invasive lithium disilicate occlusal veneers. Int J Prosthodont.

[24] Boitelle P, Mawussi B, Tapie L, Fromentin O (2014). A systematic review of CAD/CAM fit restoration evaluations. J Oral Rehabil.

[25] Hamza TA, Ezzat HA, El-Hossary MM, Katamish HA, Shokry TE, Rosenstiel SF (2013). Accuracy of ceramic restorations made with two CAD/CAM systems. J Prosthet Dent.

[26] Quintas AF, Oliveira F, Bottino MA (2004). Vertical marginal discrepancy of ceramic copings with different ceramic materials, finish lines, and luting agents: an in vitro evaluation. J Prosthet Dent.

[27] Renne W, McGill ST, Forshee KV, DeFee MR, Mennito AS (2012). Predicting marginal fit of CAD/CAM crowns based on the presence or absence of common preparation errors. J Prosthet Dent.

[28] Hasanzade M, Aminikhah M, Afrashtehfar KI, Alikhasi M (2021). Marginal and internal adaptation of single crowns and fixed dental prostheses by using digital and conventional workflows: a systematic review and meta-analysis. J Prosthet Dent.

[29] Hmaidouch R, Neumann P, Mueller WD (2011). Influence of preparation form, luting space setting and cement type on the marginal and internal fit of CAD/CAM crown copings. Int J Comput Dent.

[30] Kale E, Seker E, Yilmaz B, Özçelik TB (2016). Effect of cement space on the marginal fit of CAD-CAM-fabricated monolithic zirconia crowns. J Prosthet Dent.

[31] Webb EL, Murray HV, Holland GA, Taylor DF (1983). Effects of preparation relief and flow channels on seating full coverage castings during cementation. J Prosthet Dent.

[32] Berger L, Förtsch F, Kretschmer RR, Sednyev O, Zorzin JI, Wichmann M, Matta RE (2025). Influence of the milling strategy on the marginal fit of chairside-fabricated lithium disilicate crowns. Materials (Basel).

